# Longitudinal Metabolomics Reveals Ornithine Cycle Dysregulation Correlates With Inflammation and Coagulation in COVID-19 Severe Patients

**DOI:** 10.3389/fmicb.2021.723818

**Published:** 2021-12-03

**Authors:** Tao Li, Nianzhi Ning, Bo Li, Deyan Luo, Enqiang Qin, Wenjing Yu, Jianxin Wang, Guang Yang, Nan Nan, Zhili He, Ning Yang, Saisai Gong, Jiajia Li, Aixia Liu, Yakun Sun, Zhan Li, Tianye Jia, Jie Gao, Wang Zhang, Yanyu Huang, Jun Hou, Ying Xue, Deyu Li, Zhen Wei, Liangyan Zhang, Boan Li, Hui Wang

**Affiliations:** ^1^State Key Laboratory of Pathogens and Biosecurity, Beijing Institute of Microbiology and Epidemiology, Beijing, China; ^2^Department of Clinical Laboratory, The Fifth Medical Center of PLA General Hospital, Beijing, China

**Keywords:** COVID-19, metabolomics, inflammation, ornithine cycle, SARS-CoV-2

## Abstract

COVID-19 is a severe disease in humans, as highlighted by the current global pandemic. Several studies about the metabolome of COVID-19 patients have revealed metabolic disorders and some potential diagnostic markers during disease progression. However, the longitudinal changes of metabolomics in COVID-19 patients, especially their association with disease progression, are still unclear. Here, we systematically analyzed the dynamic changes of the serum metabolome of COVID-19 patients, demonstrating that most of the metabolites did not recover by 1–3 days before discharge. A prominent signature in COVID-19 patients comprised metabolites of amino acids, peptides, and analogs, involving nine essential amino acids, 10 dipeptides, and four N-acetylated amino acids. The levels of 12 metabolites in amino acid metabolism, especially three metabolites of the ornithine cycle, were significantly higher in severe patients than in mild ones, mainly on days 1–3 or 4–6 since onset. Integrating blood metabolomic, biochemical, and cytokine data, we uncovered a highly correlated network, including 6 cytokines, 13 biochemical parameters, and 49 metabolites. Significantly, five ornithine cycle-related metabolites (ornithine, N-acetylornithine, 3-amino-2-piperidone, aspartic acid, and asparagine) highly correlated with “cytokine storms” and coagulation index. We discovered that the ornithine cycle dysregulation significantly correlated with inflammation and coagulation in severe patients, which may be a potential mechanism of COVID-19 pathogenicity. Our study provided a valuable resource for detailed exploration of metabolic factors in COVID-19 patients, guiding metabolic recovery, understanding the pathogenic mechanisms, and creating drugs against SARS-CoV-2 infection.

## Introduction

As a new pandemic disease, coronavirus disease 2019 (COVID-19) is caused by severe acute respiratory syndrome coronavirus 2 (SARS-CoV-2) (Zhu et al., 2020). As of June 10, 2021, there have been 174,061,995 confirmed cases of COVID-19, including 3,758,560 deaths, reported to the World Health Organization.^1^ SARS-CoV-2 mainly infects the lower respiratory tract and lungs; however, many other organs, including liver, kidney, muscle, and lymph nodes, as well as gastrointestinal organs and central nervous system organs, have also been found or proposed to be attacked by this virus. The disease is associated with multiple symptoms, including fever, persistent dry cough, shortness of breath, chills, muscle pain, headache, loss of taste or smell, and gastrointestinal symptoms (Laing et al., 2020; Wu and McGoogan, 2020). Most of the infected (81%) are classified as mild or moderate COVID-19 patients, who usually recover with, or even without, conventional medical treatment. However, about 19% of confirmed patients develop severe pneumonia or multiple organ failure with high mortality (Wu and McGoogan, 2020).

In recent years, patient-based immunological and inflammatory profile studies have widely been used to identify the host response or signatures of many infectious diseases, including COVID-19 (Harapan et al., 2020; Laing et al., 2020; Song et al., 2020b; Thomas et al., 2020). It has been reported that the levels of many inflammatory markers, such as interleukin (IL)-6, IL-7, monocyte chemoattractant protein (MCP)-1, and macrophage inflammatory protein (MIP)-1α, are elevated in COVID-19 patients (Ciaccio and Agnello, 2020). Inflammatory cytokine signatures, such as IL-6 and IL-8, could be used to predict COVID-19 severity and survival (Del Valle et al., 2020). Metabolomics, which identifies and quantifies small-molecule metabolites, is the omics closest to clinical phenotypes (Ayres, 2020). Interestingly, many metabolites have a potent biological effect on critical pathophysiological processes, which reflect the metabolic activity of tissues and influence the clinical phenotype. The best approach to characterize the metabolic changes of complex syndromes such as COVID-19 is to perform high-throughput untargeted studies in large series of patients. Several studies about the metabolome of COVID-19 patients have exhibited the metabolic disorders and the potential markers differentiating healthy subjects and COVID-19 patients, or severe and non-severe cases (Shen et al., 2020; Song et al., 2020a). These discoveries have highlighted the dysregulation of multiple immune and metabolic components in clinically severe patients. However, the longitudinal changes of metabolomics in the serum of mild and severe COVID-19 patients, especially the metabolic status during the recovery stage, are unclear.

In the previous reports, amino acid metabolism dysregulation has been observed in COVID-19 patients; for example, there is altered tryptophan metabolism in the kynurenine pathway, which regulates inflammation and immunity in COVID-19 patients (Thomas et al., 2020). The ornithine cycle (urea cycle) is a critical amino acid metabolism pathway by which mammals dispose of waste nitrogen (Morris, 2002). Ammonia produced by protein and amino acid metabolism in the body combines with ornithine and CO_2_ through a five-step enzymatic reaction to produce ornithine and urea. Ornithine enters mitochondria and participates in the ornithine cycle again, while urea is excreted *via* urine. The whole process of the ornithine cycle occurs in the liver (Morris, 2002). The ornithine cycle is closely related to other metabolic processes. For example, in the process of the tricarboxylic acid cycle (TCA), oxaloacetic acid produced by the TCA is converted to aspartic acid, which provides an important nitrogen source for urea production in the ornithine cycle (Posset et al., 2019). Ornithine cycle dysregulation plays an important role in regulating several metabolic processes, leading to hyperornithinemia, hyperammonemia, and gyrate atrophy in humans (Sivashanmugam et al., 2017). Ornithine cycle dysregulation serves as a biomarker in cancer patients’ biofluids and is associated with an enhanced response to immune checkpoint therapies (Lee et al., 2018). However, the changes in ornithine cycle metabolism, especially the association with disease progression, have rarely been studied in COVID-19 patients.

To address these questions, we systematically analyzed the dynamic changes of the serum metabolome in COVID-19 patients, including the initial three stages of onset symptoms (1–3, 4–6, and 7–9 days since onset) and 1–3 days before discharge. A significant feature observed in COVID-19 patients is the significant regulation of amino acid metabolism, in particular the metabolites of the urea cycle. We also detected the dynamic changes in multiple cytokines and biochemical parameters to observe the immune response and tissue dysfunction during the progression of COVID-19. We analyzed the correlations between the metabolites and these clinical variables, and discovered a highly correlated network consisting of 6 cytokines, 13 biochemical parameters, and 49 metabolites. Significantly, five ornithine cycle-related metabolites highly correlated with “cytokine storms” and coagulation index. These results suggest that ornithine cycle dysregulation may be a potential pathogenetic factor of COVID-19, and may serve as an important target for regulating metabolic disorders in COVID-19 patients.

## Materials and Methods

### Patients and Samples

Our team procured serum samples from 47 COVID-19 patients at the Fifth Medical Center of PLA General Hospital. They were diagnosed as COVID-19 according to the Chinese Government Diagnosis and Treatment Guideline (Trial 5th version) (National Health Commission of the PRC [NHCPRC], 2020). According to the guideline, COVID-19 patients are classified into four subgroups: Mild: mild symptoms without pneumonia; Moderate: fever or respiratory tract symptoms with pneumonia; Severe: fulfill any of the three criteria: respiratory distress, respiratory rate ≥30 times/min; means oxygen saturation ≤93% in resting state; arterial blood oxygen partial pressure/oxygen concentration ≤300 mmHg (1 mmHg = 0.133 kPa); Critical: fulfill any of the three criteria: respiratory failure and require mechanical ventilation; shock incidence; admission to ICU with other organ failure (Shen et al., 2020). In this study, the mild group (*n* = 30) included mild and moderate cases, and the severe group (*n* = 17) had severe and critical cases. The admission data of these patients were collected and checked independently by two physicians. We also collected serum samples from 20 healthy individuals as control. Clinical and demographic data of the 47 COVID-19 patients (Supplementary Table 1) were in good agreement with the previous report on clinical characteristics of COVID-19 in China (Guan et al., 2020). There were no significant differences between the ages or sexes of the COVID-19 patients and the controls (Supplementary Table 2).

All the serum samples were collected as whole venous blood in the early morning before diet using serum separation tubes. The blood samples were centrifuged at 3,500 rpm for 10 min, and the supernatants were collected as serum samples. The serum samples were frozen at −80^°^C. The study was performed following the Declaration of Helsinki principle for ethical research. Ethics Committee approved the study protocol of the Fifth Medical Center of PLA General Hospital. Written informed consent was waived by the Ethics Committee of the designated hospital for emerging infectious disease.

### Metabolites Extraction

The method of metabolites extraction is based on previous report (Dunn et al., 2011). The sample (200 μl) was transferred to an EP tube. After the addition of 1 μl isotopically labeled internal standard mixture, the samples were vortexed for 60 s. Then, the sample was centrifuged at 12,000 rpm for 15 min at 4^°^C. The resulting supernatant was transferred to a fresh glass vial for analysis.

### LC-MS/MS Analysis

LC-MS/MS analyses were performed using a UHPLC system (Vanquish, Thermo Fisher Scientific) with a UPLC BEH Amide column (2.1 mm × 100 mm, 1.7 μm) coupled to Q Exactive HFX mass spectrometer (Orbitrap MS, Thermo Fisher Scientific). The mobile phase consisted of 25 mmol/L ammonium acetate and 25 ammonia hydroxide in water (pH = 9.75) (A) and acetonitrile (B). The analysis was carried out with elution gradient as follows: 0–0.5 min, 95% B; 0.5–7.0 min, 95–65% B; 7.0–8.0 min, 65–40% B; 8.0–9.0 min, 40% B; 9.0–9.1 min, 40–95% B; 9.1–12.0 min, 95% B. The column temperature was 30^°^C. The auto-sampler temperature was 4^°^C, and the injection volume was 2 μl.

The QE HFX mass spectrometer was used for its ability to acquire MS/MS spectra on information-dependent acquisition (IDA) mode in the control of the acquisition software (Xcalibur, Thermo Fisher Scientific) (Smith et al., 2006). In this mode, the acquisition software continuously evaluates the full scan MS spectrum. The ESI source conditions were set as follows: sheath gas flow rate as 50 Arb, Aux gas flow rate as 10 Arb, capillary temperature 320^°^C, full MS resolution as 60,000, MS/MS resolution as 7,500, collision energy as 10/30/60 in NCE mode, spray voltage as 3.5 kV (positive) or -3.2 kV (negative), respectively.

### Data Preprocessing and Annotation

The raw data were converted to the mzXML format using ProteoWizard and processed with an in-house program, which was developed using R and based on XCMS, for peak detection, extraction, alignment, and integration. Then, an in-house MS2 database (BiotreeDB) (Xie et al., 2021) was applied in metabolite annotation.

### Quality Control of Metabolome Analysis

The quality control (QC) samples were prepared by mixing all the samples in equal volume (10 μl) (Dunn et al., 2011). The QC samples were as a technical replicate that was run 20 times throughout the experiment, and extracted water samples served as blanks. A mixture of internal standards was also spiked into every sample to aid chromatographic peak alignment and instrument stability monitoring. Instrument variability was determined by calculating the median relative SD (RSD) of all internal standards in each sample. The experimental process variability was determined by calculating the median RSD for all endogenous metabolites present in the pooled quality control samples.

### Luminex Multiplex Cytokine Test

The levels of 34 cytokine molecules were measured using Luminex based bead array, the ProcartaPlex Hu Cytokine/Chemokine Paneel 1A 34plex (Invitrogen, Thermo Fisher Scientific), for the following biomarkers: IFN-γ, IL-12p70, IL-13, IL-1β, IL-2, IL-4, IL-5, IL-6, TNF-α, GM-CSF, IL-18, IL-10, IL-17A, IL-21, IL-22, IL-23, IL-27, IL-9, IFN-α, IL-31, IL-15, IL-1α, IL-1RA, IL-7, TNF-β, Eotaxin, GRO-α, IL-8, IP-10, MCP-1, MIP-1α, MIP-1β, SDF-1α, and RANTES. In brief, 25 μl serum samples were tested for the levels of the cytokines, using the multiplex immunoassay containing fluorescent-labeled beads conjugated with a specific monoclonal antibody for the target molecule, according to the manufacturer’s recommendations (Montoya et al., 2017; Stanjek-Cichoracka et al., 2019). Appropriate standards included 34 beads and quality control (provided by the manufacturers with the test sample plate). The software determines and interprets the data and concentrations of analytes in samples determined from the standards run in the test. Samples were analyzed using the standards that were run in each of the plates. Each run contained appropriate quality controls run in duplicates, and results were interpreted from the standard using Luminex 200 system.

### Statistical Analysis

Patient characteristics were summarized using the standard descriptive statistics: median/IQR for continuous variables and count/percent for categorical variables. The *t*-test was conducted to compare values measured in the COVID-19 patients against controls, and the severe COVID-19 patients against mild patients’ values. Metabolites contributing were calculated according to the variable importance for project (VIP) values (<1.0) and *p*-values after FDR (<0.05) (Fu et al., 2020; Xie et al., 2021). The correlation of each metabolite with the biochemical parameters or cytokines was analyzed using the non-parametric Spearman coefficient of rank correlation.

## Results

### Sample Collection, Study Design, and Overview of Blood Metabolomic Changes

**FIGURE 1:**
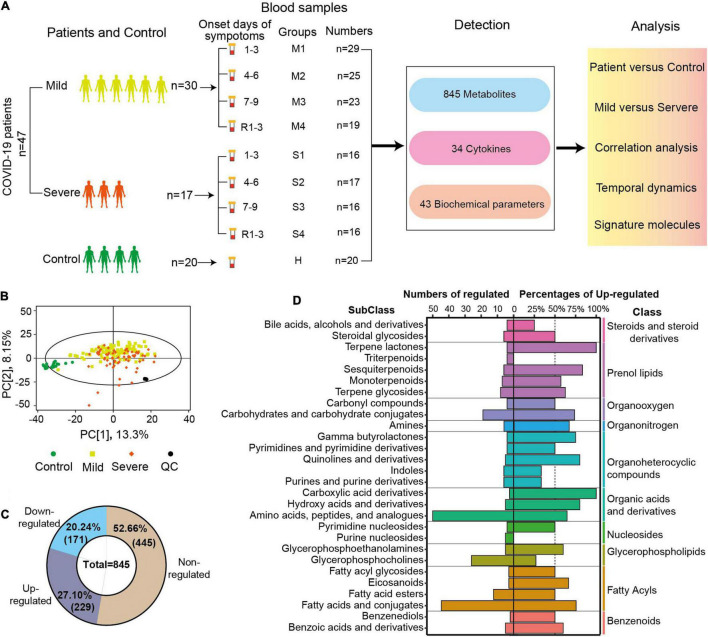
Sample collection, study design, and overview of blood metabolomic changes. **(A)** Overview of the blood sample collection and study design from COVID-19 patients and controls. The serum samples of mild patients, severe patients, and controls were collected at four time points after diagnosis and before discharge. R1–3 refers to the samples collected 1–3 days before discharge. First, second, third, and fourth samples of severe (mild) cases and control samples were indicated by S1, S2, S3, S4 (M1, M2, M3, M4), and C. A total of 845 metabolites, 34 cytokines, and 43 biochemical parameters of these samples were detected, and these data were compared and correlation was analyzed. **(B)** Principal component analysis (PCA) plot of the samples from COVID-19 patients, controls, and quantity control (QC) using the metabolomic data. Each dot represents an individual sample. **(C)** The numbers and percentages of non-regulated, downregulated, or upregulated metabolites in the samples from COVID-19 patients compared with controls (FDR *p* < 0.05, VIP > 1). **(D)** The diagram shows the classification of metabolites with significant differences between patients and controls. The numbers of significantly changed metabolites in different subclasses are shown on the left, and the percentages of upregulated metabolites among the total metabolites are indicated on the right (FDR *p* < 0.05, VIP > 1.0). The classes of those metabolites are also shown in the right panel.

In total, 161 blood samples were collected from 47 patients in the Fifth Medical Center of PLA General Hospital (Figure 1A). Serial samples were obtained throughout COVID-19 hospitalization, including three post-diagnosis time periods (1–3, 4–6, and 7–9 days since onset) and one recovery time period [1–3 days before discharge (R1–3)]. Twenty blood samples from 20 healthy volunteers were used as control. The samples were transported to the laboratory and processed for metabolomics and other analyses. An untargeted metabolomics approach based on liquid chromatography-mass spectrometry (LC-MS) was used to observe the dynamic metabolic profile of COVID-19 patients and identify the metabolic characteristics associated with severe disease. In total, 845 metabolites (excluding 55 drugs and their metabolites) with over 6,500 LC-MS peaks were identified and quantified, covering 97 classes and 187 subclasses. Analyzing the metabolomes by principal component analysis (PCA), there was a noticeable distinction between the samples of patients and those of controls, while there was a partial overlap between the samples of severe and mild cases (Figure 1B). The quality control (QC) samples were prepared by mixing an equal aliquot from all of the samples. Twenty tests of the QC samples throughout the experiment showed a high degree of consistency and reproducibility.

Comparing the metabolomic data between COVID-19 patients and controls, the levels of 400 metabolites (47.34%) were significantly different (FDR *p* < 0.05, VIP > 1.0, Figure 1C). All or most of the metabolites in indoles (100%), terpene lactones (100%), fatty acid esters (80%), and pyrimidine nucleosides (80%) were significantly regulated in COVID-19 patients (FDR *p* < 0.05, VIP > 1.0, Supplementary Figure 1). In COVID-19 patients, 229 metabolites (27.10%) were significantly upregulated, and 171 metabolites (20.24%) were significantly downregulated (Figure 1C). The majority of the metabolites of three classes (prenol lipids, organonitrogen, organic acids, and derivatives) were upregulated in the patients’ samples. More than 50% of the metabolites were upregulated in 15 subclasses and downregulated in six subclasses. All of the altered metabolites in carboxylic acid derivatives and terpene lactones were upregulated in COVID-19 patients, while all of the altered metabolites in triterpenoids and purine nucleosides were downregulated (Figure 1D). Amino acids, peptides, and analogs (50 metabolites, 64% upregulated), and fatty acids and conjugates (45 metabolites, 75.56% upregulated) were the two biggest subclasses of the regulated metabolites (Figure 1D). The metabolite analysis at the subclass levels showed a complete and high-resolution picture of the metabolic regulation in COVID-19 patients.

### Different Subclasses of Metabolites Have Special Characteristics of Metabolic Dynamic Profiles

**FIGURE 2:**
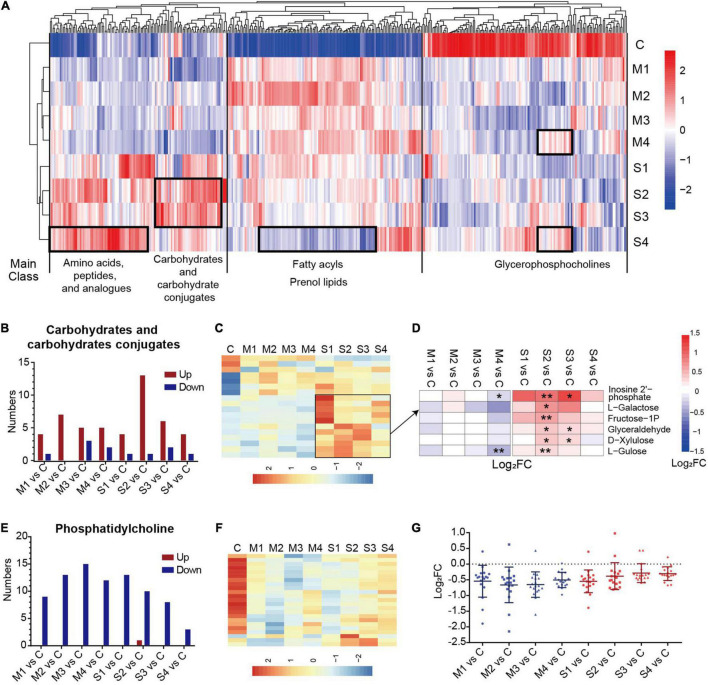
The significantly regulated metabolites in carbohydrates and carbohydrate conjugates and phosphatidylcholine in COVID-19 patients. **(A)** The hierarchical clustering analysis of the levels of 400 significantly regulated metabolites in the blood samples from mild or severe patients at four sampling times (M1, M2, M3, M4, S1, S2, S3, or S4) and controls **(C)**. **(B)** The numbers of upregulated or downregulated metabolites in carbohydrates and carbohydrates conjugates **(B)** and phosphatidylcholine **(E)** for mild or severe patients at four sampling times (M1, M2, M3, M4, S1, S2, S3, or S4) compared with controls **(C)**. The heat map of hierarchical clustering analysis of significantly regulated metabolites in carbohydrates and carbohydrate conjugates **(C)**, and phosphatidylcholine **(F)**. **(D)** The heat map of the fold changes (FC) of the representative metabolites in carbohydrates and carbohydrates conjugates and associated *p*-values for COVID-19 patients compared with controls. **p < 0.01, *p < 0.05. **(G)** The fold changes (FC) of all regulated phosphatidylcholines for COVID-19 patients compared with controls. Each dot represents phosphatidylcholine. The mean value and standard deviation of Log_2_FC are also marked in the figure.

Moreover, the dynamic changes of the serum metabolomics through the course of COVID-19 were analyzed. The numbers of significantly changed metabolites in most subclasses were similar at the four sampling times, and were only slightly reduced in M4 or S4 (Supplementary Figure 2), meaning that most of the metabolites of COVID-19 patients did not recover at 1–3 days before discharge. Hierarchical clustering analysis based on the levels of metabolites at the four sampling times and control showed that the dysregulated metabolites in different subclasses had obviously different dynamic expression profiles (Figure 2A). For example, most glycerophosphocholines were downregulated and apparently recovered before discharge (M4 or S4). In contrast, most fatty acyls and prenol lipids were upregulated and did not recover at 1–3 days before hospital discharge in mild patients (M4).

A prominent signature observed in COVID-19 patients involved the metabolites in amino acids, peptides, and analogs, which is carefully described later in the text. The carbohydrates and carbohydrate conjugates showed a high proportion of upregulated metabolites at all four sampling periods, with the highest numbers of upregulated metabolites at stage S2 (Figure 2B). The levels of the majority of upregulated metabolites in the subclass of severe cases were higher (Figure 2C). These metabolites, such as L-galactose and L-gulose, were significantly different mainly at the S2 or S3 stages in comparison with the controls (Figure 2D). These results suggested that dysregulation of carbohydrate metabolism may be associated with the disease severity in COVID-19 patients. Interestingly, the levels of all regulated phosphatidylcholines (except one metabolite in S2) were significantly lower in mild and severe patients than in controls. Specifically, the number of differential phosphatidylcholines was obviously reduced in the samples at S4 (Figure 2E). The differences between the samples from COVID-19 patients 1–3 days before discharge (M4 or S4) and controls were greatly decreased (Figures 2F,G). These results showed that the metabolites of phosphatidylcholine evidently recovered before the discharge of COVID-19 patients, especially in severe cases.

### Amino Acid Metabolism, Especially the Ornithine Cycle, Is Significantly Dysregulated in Severe Patients

The Kegg pathway analysis showed that 11 metabolic pathways were significantly dysregulated in COVID-19 patients, among which seven pathways were related to amino acid metabolism (Supplementary Figure 3, *p* < 0.05). Three pathways (alanine, aspartate, and glutamate metabolism; arginine and proline metabolism; phenylalanine metabolism) were significantly dysregulated in the early stages of severe COVID-19 patients. The other two pathways (glutamine and glutamate metabolism; glycine, serine, and threonine metabolism) were considerably dysregulated in all four sampling times of mild and severe COVID-19 patients, and did not recover before discharge. These results showed that amino acid metabolic disorders deserve serious attention, especially in severe cases.

**FIGURE 3:**
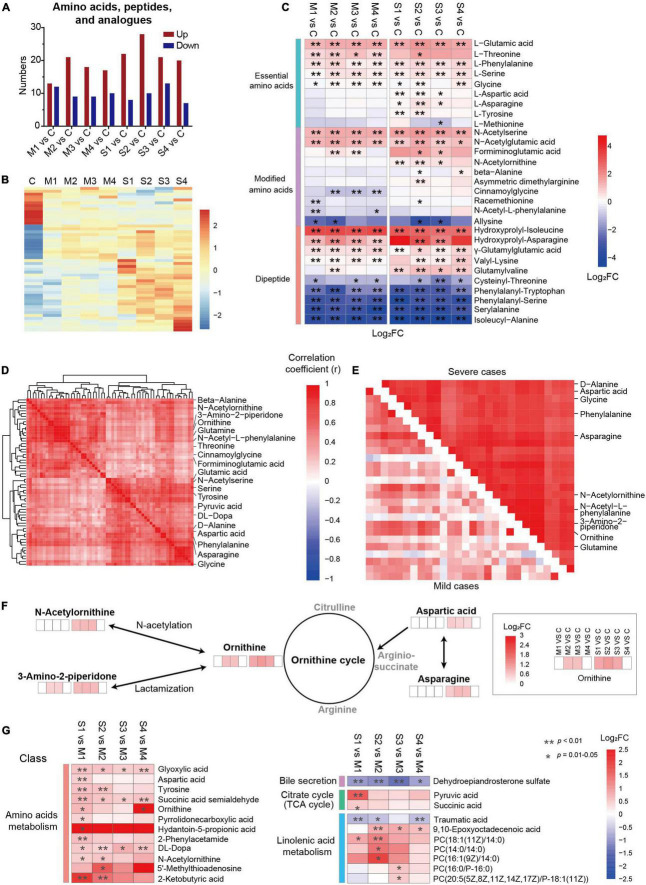
Dysregulation of amino acid metabolism, especially the metabolites related to the ornithine cycle, in severe COVID-19 patients. **(A)** The numbers of upregulated or downregulated metabolites in amino acids, peptides, and analogs in mild and severe patients at four sampling times (M1, M2, M3, M4, S1, S2, S3, or S4) compared with controls **(C)**. **(B)** The heat map of hierarchical clustering analysis of significantly regulated metabolites in amino acids, peptides, and analogs. **(C)** The heat map of the fold changes (FC) of the representative metabolites in amino acids, peptides, and analogs and associated *p*-values for COVID-19 patients compared with controls. **p < 0.01, *p < 0.05. **(D)** A group of closely correlated metabolites primarily associated with amino acid metabolism. **(E)** The metabolites of amino acids, peptides, and analogs with higher correlation coefficients in severe patients than in mild ones. **(F)** The amino acids and derivatives of the ornithine cycle were significantly different between COVID-19 patients and controls. **(G)** The significantly differential metabolites between mild and severe patients. The enrichment pathways are also shown on the left panel. **p < 0.01, *p < 0.05.

The metabolites in amino acids, peptides, and analogs, involving essential amino acids, modified amino acids, and dipeptides, were significantly regulated (Figures 3A–C). The number of upregulated metabolites in this subclass was the largest at S2 (Figure 3A), and the levels of nearly half of the metabolites were higher in severe cases (Figure 3B). Interestingly, most of the regulated essential amino acids (except methionine) were significantly upregulated in COVID-19 patients compared with controls (Figure 3C). In contrast, the levels of four dipeptides (phenylalanyl-tryptophan, phenylalanyl-serine, serylalanine, and isoleucyl-alanine) were significantly lower in mild and severe patients at all four sampling times. Moreover, the majority of dysregulated dipeptides did not recover until discharge. Four N-acetylated amino acids (N-acetylserine, N-acetylglutamic acid, N-acetylornithine, and N-acetyl-L-phenylalanine) were significantly regulated in COVID-19 patients, suggesting that N-acetylation might be involved in the mechanism of SARS-CoV-2 infection. These results showed that different types of amino acids, peptides, and analogs might be regulated in different ways in COVID-19 patients. In addition, the roles of dipeptides and modified amino acids in SARS-CoV-2 infection need to be studied in the future.

The association analysis of the dysregulated metabolites showed a close cluster with 61 metabolites, including 12 amino acids and six modified amino acids (Figure 3D), which may play essential roles in amino acid metabolic disorders in SARS-CoV-2 infection. Among them, 27 metabolites formed a subcluster, which had a higher correlation coefficient in severe patients than in mild ones, including five ornithine cycle-related metabolites (ornithine, N-acetylornithine, 3-amino-2-piperidone, aspartic acid, and asparagine) (Figure 3E and Supplementary Figure 4). In the ornithine cycle, ornithine is the direct substrate; asparagine can be converted into aspartic acid to participate in the ornithine cycle; and N-acetylornithine and 3-amino-2-piperidone are N-acetylation and delta-lactam derivatives of ornithine (Figure 3F). Interestingly, all five metabolites were significantly upregulated mainly in severe patients, and recovered before discharge. These data indicated that the ornithine cycle was considerably dysregulated in patients with severe cases of COVID-19.

The levels of dysregulated metabolites were compared between severe and mild patients (Figure 3G). In total, the levels of 152 metabolites were significantly different between severe and mild patients, including 43 metabolites in M1 vs. S1, 65 metabolites in M2 vs. S2, 32 metabolites in M3 vs. S3, and 69 metabolites in M4 vs. S4 (FDR *p* < 0.05, VIP > 1.0, Supplementary Figure 5A). Five metabolites (3-hydroxyisovalerylcarnitine, succinic acid semialdehyde, dehydroepiandrosterone sulfate, glyoxylic acid, and DL-dopa) were significantly different between severe and mild patients at all four stages (FDR *p* < 0.05, Supplementary Figure 5B), which indicates that they could be essential candidate molecules to distinguish between mild and severe patients at any time point. The average levels of 12 amino acid metabolism-related metabolites (such as tyrosine), seven linolenic acid metabolism-related metabolites [such as PC (18:1(11Z)/14:0)], two citrate cycle-related metabolites (such as pyruvic acid), and one bile secretion-related metabolite (such as dehydroepiandrosterone sulfate) were significantly different between mild and severe patients (FDR *p* < 0.05, Figure 3G). Specifically, three metabolites (ornithine, N-acetylornithine, and aspartic acid) of the ornithine cycle were significantly higher in severe patients than in mild ones, mainly at days 1–3 or 4–6 since onset (S1 vs. M1, or S2 vs. M2, FDR *p* < 0.05, Figure 3G). According to all these results, amino acid metabolism is highly imbalanced in SARS-CoV-2 infection, especially in the early stages of severe patients. In particular, ornithine cycle dysregulation is a critical element of amino acid metabolic disorders.

### Ornithine Cycle-Related Metabolites Are Strongly Associated With “Cytokine Storms” and Coagulation Index in Severe Patients

In addition to metabonomics, 34 cytokines and 44 biochemical parameters were also systematically detected and analyzed in the serum of COVID-19 patients. The levels of 11 cytokines in COVID-19 patients were significantly higher than those in controls at one or more sampling times (Supplementary Figure 6A, *p* < 0.05). The fold changes (FC) of most cytokines compared with controls were greatly reduced at the recovery stage (1–3 days before discharge). A significant difference was observed in the levels of IL-6 and IL-7 between severe and mild patients at 1–3 days and 4–6 days since onset (Supplementary Figure 6B, *p* < 0.05 or *p* < 0.01), which is in accordance with the published literature about IL-6 as a severity predictor of COVID-19. Eighteen biochemical parameters were significantly different between severe and mild patients at 1–9 days since onset (Supplementary Figure 7), showing that multiple organ injury occurred during the infection in severe patients. These results demonstrated that the severity of host inflammation responses and tissue dysfunction in COVID-19 patients mainly occurred at the early stage of infectious symptoms, and recovered before discharge.

**FIGURE 4:**
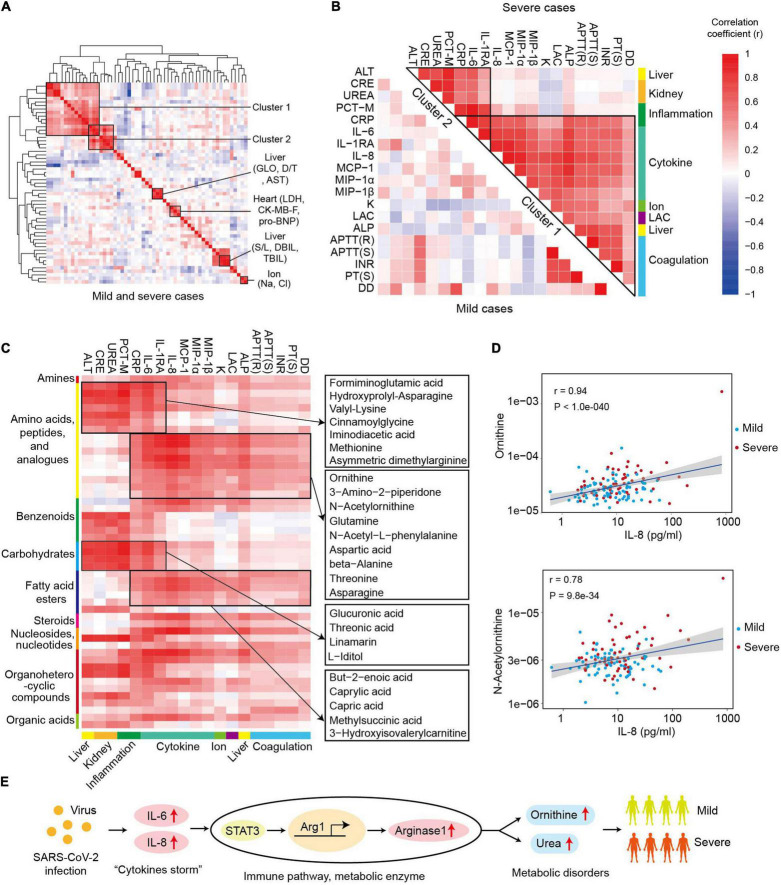
Ornithine cycle-related metabolites highly associated with “cytokine storms” and coagulation index in severe patients. **(A)** The heat map of correlation coefficients between different cytokines and biochemical parameters in COVID-19 patients. LDH, lactate dehydrogenase; CK-MB-F, luminescent creatine kinase isoenzyme MB; pro-BNP, pro brain natriuretic peptide; GLO, globulin; AST, aspartate aminotransferase; D/T, direct bilirubin/total bilirubin. **(B)** The heat map of correlation coefficients among different items of clusters 1 and 2 in mild or severe samples. **(C)** The heat map of correlation coefficients between cluster 1 (or cluster 2) and metabolomics. The representative metabolites are shown in the right panel. **(D)** The correlation plots between IL-8 and ornithine or N-acetylornithine. Correlation coefficient (r) and *p*-values are indicated at the top right of each comparison. **(E)** The role and possible mechanism of ornithine in SARS-COV-2 infection. SARS-CoV-2 infection activates “cytokine storm” (IL-6 or IL-8), which induces overexpression of arginase 1 and excessive ornithine in immune cells. The levels of ornithine and its derivatives positively correlate with the symptoms in severe patients.

The correlation map of cytokines and biochemical parameters showed multiple clusters, including ion-related cluster (Na^+^, Cl^+^), heart-related cluster (LDH, CK-MB-F, pro-BNP), and liver-related clusters (GLO, AST, D/T) (Figure 4A). Notably, there were also two clusters with the items related to different tissues, such as cluster 1 and cluster 2. Cluster 1 contained a total of seven variables representing the inflammation (IL-6, IL-1RA, CRP, and PCT-M), renal function (UREA and CRE), and hepatic function (ALT), which indicated the potential association between inflammation response, alanine aminotransferase, and disorder of renal function in the progression of COVID-19. Cluster 2 comprised 15 variables related to coagulation (APTT (R), APTT (S), INR, PT (S), DD), cytokines (IL-6, IL-8, IL-1RA, MCP-1, MIP-1α, MIP-1β), acid–base balance (LAC), ion (K^+^), liver (alkaline phosphatase, ALP), and C-reactive protein (CRP) (Figure 4B). The correlation coefficients of the two clusters were higher in severe samples. Primarily, CRP and IL-6 were highly associated with the majority items of cluster 1 and cluster 2 in severe cases (*r* > 0.5, Figure 4B). All of these results showed the important roles of “cytokine storms” and inflammation response in COVID-19 progression.

Furthermore, the global correlation between cytokines or biochemical parameters and metabolites in COVID-19 patients was systematically analyzed so as to identify the potential role of metabolites in SARS-CoV-2 infection (Figure 4C). The levels of the items in the cluster 1 and cluster 2 were strongly associated with the metabolomic changes in COVID-19 patients. There were 49 metabolites significantly correlated with the levels of the items in cluster 1 or cluster 2, including 16 metabolites in amino acids, peptides, and analogs (*r* > 0.4, *p* < 0.01, Figure 4C). Moreover, cluster 1 and cluster 2 were related to two groups of differentially expressed metabolites. In cluster 1, seven amino acids, peptides, and analogs; four carbohydrates and analogs; two benzenoids; two organoheterocyclic compounds; and one nucleoside, nucleotide, and analog were significantly related with all the items. Specifically, some metabolites [such as formiminoglutamic acid (Luhby et al., 1959) and cinnamoylglycine (Ganti et al., 2012)] significantly correlated with most items of cluster 1 and cluster 2. In cluster 2, amino acids, peptides, and analogs, and fatty acid esters were the two main classes of correlated metabolites, and most of the related metabolites had a higher correlation coefficient (*r*) with the parameters of cluster 2 in the samples from severe patients (Supplementary Figure 8). Interestingly, six ornithine cycle-related metabolites (ornithine, N-acetylornithine, 3-amino-2-piperidone, glutamine, aspartic acid, and asparagine) were highly associated with the items of cluster 2, for example, ornithine and IL-8 (*r* = 0.94, Figure 4D). Thus, we inferred that ornithine cycle dysregulation might play essential roles in the severity of COVID-19 patients, which needs to be further studied.

## Discussion

In this study, we obtained the comprehensive dynamic profiles of blood parameters, including metabolomes, biochemical parameters, and cytokines, during different stages of COVID-19 progression. We found that nearly half of the metabolites were significantly dysregulated during the disease process, which is in good agreement with previous reports on the metabolic disorders in COVID-19 patients (Shen et al., 2020; Song et al., 2020a). The longitudinal changes of metabolomics, especially the patient’s metabolic status at discharge, were uncovered in this study. The dysregulated metabolites in different classes showed obviously different dynamic expression profiles. For example, carbohydrates and carbohydrate conjugates had a high proportion of upregulated metabolites, while almost all phosphatidylcholines were downregulated. The level changes and numbers of dysregulated metabolites in most classes were highest at S2 or S3, which is in agreement with the peak symptoms in COVID-19 patients. These results provide valuable metabolic disorder information, which can help doctors to select the appropriate time to supplement specific nutrients or administer drugs to restore patients’ metabolic balance. Our results showed that the levels of most cytokines (Supplementary Figure 6) and biochemical parameters (Supplementary Figure 7) normalized with time. In contrast, most metabolites (such as amino acids, peptides, and analogs) did not recover by 1–3 days before discharge, different from other blood parameters (such as “cytokine storms”). Our results indicated that the metabolic disorders of COVID-19 patients might have a long road to recovery even after leaving the hospital. A previous report showed that metabolic abnormalities were detected in people with SARS-CoV-2 infection 12 years after infection (Wu et al., 2017). It is interesting that most cytokines and biochemical parameters, but not the metabolites, returned to normal, which may suggest the role and possible mechanism of metabolites in “long COVID.” Taken together, our results demonstrated that the metabolic status of COVID-19 patients requires long-term attention, and metabolic parameters, especially at the early stage of infection, have to be managed to control the disease course of COVID-19 (Ayres, 2020).

A prominent signature of the metabolic disorder in COVID-19 patients was amino acid metabolism dysregulation, which has been observed in previous reports (Shen et al., 2020; Thomas et al., 2020). In this study, we revealed several novel aspects of amino acid metabolism dysregulation in COVID-19 patients. First, two types of amino acid-related metabolites (modified amino acids and dipeptides) were significantly regulated in COVID-19 patients. Although their functions and mechanisms are not known, multiple modified amino acids have been reported as markers for clinical diagnoses in many diseases, such as formiminoglutamic acid in clinical folic acid deficiency (Luhby et al., 1959). Four dysregulated N-acetylated amino acids, previously identified in patients with inborn errors of amino acid metabolism (Jellum et al., 1986), were found in COVID-19 patients in this study. Interestingly, the levels of 10 dipeptides, whose functions and applications have been poorly studied (Yagasaki and Hashimoto, 2008), were significantly altered in COVID-19 patients. The role and mechanism of modified amino acids and dipeptides in COVID-19 patients require further study.

Furthermore, we obtained a comprehensive correlation map of the metabolites, cytokines, and biochemical parameters in COVID-19 patients. The results highlighted the close associations among the inflammatory response (including cytokines CRP and PCT-M), the dysfunction of tissues (such as liver and kidney), the coagulation index [such as APTT (R), INR, and PT (S)], and the dynamic changes of metabolic profiles (especially for amino acids, peptides, and analogs) in the COVID-19 progression, especially for severe patients. COVID-19 is a complex, multisystem condition (Lauretani et al., 2020; Simpson and Newburger, 2020), and a holistic understanding is necessary to maximize survival (Ayres, 2020). More importantly, the 49 highly correlated metabolites in the network, including 16 amino acids, peptides, and analogs, may act as critical regulators mediating inflammation responses and pathological changes in COVID-19 patients.

The ornithine cycle (urea cycle) is a critical amino acid metabolism pathway, and its dysregulation has been observed in many diseases, such as infection (Lercher et al., 2019), cancer (Lee et al., 2018), and hypertension (Zheng et al., 2018). In this study, we found that the ornithine cycle was significantly upregulated in SARS-CoV-2 infection. Five ornithine cycle-metabolites (ornithine, N-acetylornithine, 3-amino-2-piperidone, aspartic acid, and asparagine) positively correlated with multiple cytokines and coagulation index in COVID-19 patients, especially in severe cases. Excessive ornithine could be induced by multiple pathogens (such as *Mycobacterium tuberculosis* and hepatitis virus) (Lercher et al., 2019; Nishio and Rehermann, 2019; Sivangala Thandi et al., 2020). However, the mechanism of action behind the multifaceted role of ornithine has not yet been fully unraveled (Sivashanmugam et al., 2017). Many studies (Narita et al., 2013; Monticelli et al., 2016; Murray, 2016) have reported that Arginase 1—an enzyme catalyzing arginine to ornithine and urea—is a metabolic checkpoint in immune response and inflammation, and its expression in immune cells (such as dendritic cell and macrophages) could be activated by IL-6 (or IL-8) (Figure 4E). Lercher et al. (2019) showed that chronic viral infection-induced interferon alters the expression and function of the key enzymes of the ornithine cycle in hepatocytes, and serum levels of ornithine modulate antiviral T-cell responses and liver pathology. Thus, we infer that ornithine cycle dysregulation may also contribute to inflammatory response and organ dysfunction in COVID-19 patients. These metabolites highly associated with the symptoms could be potential candidate targets to develop effective defense strategies for patients.

## Conclusion

We found that the ornithine cycle dysregulation significantly correlates with inflammation and coagulation in severe patients, which may be a potential mechanism of COVID-19 pathogenicity. Our study provided a valuable resource for in-depth exploration of metabolic factors in COVID-19 patients, guiding metabolic recovery, understanding the pathogenic mechanisms, and creating drugs against SARS-CoV-2 infection.

## Data Availability Statement

The datasets presented in this study can be found in online repositories. The names of the repository/repositories and accession number(s) can be found in the article/Supplementary Material.

## Ethics Statement

The studies involving human participants were reviewed and approved by the Fifth Medical Center of PLA General Hospital. Written informed consent to participate in this study was provided by the participants’ legal guardian/next of kin.

## Author Contributions

HW and BAL designed the study. BAL, BL, EQ, GY, NY, AL, TJ, WZ, JH, and ZW collected the samples and information. TL, NZN, DLu, WY, JW, NN, ZH, SG, JL, and HW performed the experiment and data analysis. YS, ZL, JG, YH, DLi, LZ, and YX helped with article discussion. TL wrote the manuscript. All authors contributed to the article and approved the submitted version.

## Conflict of Interest

The authors declare that the research was conducted in the absence of any commercial or financial relationships that could be construed as a potential conflict of interest.

## Publisher’s Note

All claims expressed in this article are solely those of the authors and do not necessarily represent those of their affiliated organizations, or those of the publisher, the editors and the reviewers. Any product that may be evaluated in this article, or claim that may be made by its manufacturer, is not guaranteed or endorsed by the publisher.

## Footnotes

1
https://covid19.who.int/

## Abbreviations

COVID-19: Coronavirus disease 2019
SARS-CoV-2: Severe acute respiratory syndrome coronavirus 2
IL: Interleukin
MCP: Monocyte chemoattractant protein
MIP: Macrophage inflammatory protein
TCA: Tricarboxylic acid cycle
LC-MS: Liquid chromatography-mass spectrometry
PCA: Principal Components Analysis
QC: Quality control
LDH: Lactate dehydrogenase
CK-MB-F: Luminescent creatine kinase isoenzyme MB
pro-BNP: Pro brain natriuretic peptide
GLO: Globulin
AST: Aspartate aminotransferase
D/T: Direct bilirubin/total bilirubin
CRP: C-reactive protein
PCT-M: Procalcitonin
CRE: Creatinine
ALT: Alanine aminotransferase
APTT(R): Active part thromboplastin ratio
APTT(S): Activated partial thromboplastin time
INR: International normalized ratio
PT(S): Prothrombin time
DD: D-D dimer
LAC: Lactic acid
ALP: Alkaline phosphatase.
