# Supplementary material for: Longitudinal Metabolomics Reveals Ornithine Cycle Dysregulation Correlates With Inflammation and Coagulation in COVID-19 Severe Patients
Source: Front Microbiol. 2021 Dec 3;12:723818. doi: 10.3389/fmicb.2021.723818 (PMC8678452; doi:10.3389/fmicb.2021.723818)
Supplement: Supplementary file 1 [file Table_1.docx]

Table S1 Basic information of 30 mild and 17 severe COVID-19 patients.

|  | **Mild patients （n = 30)** | **Severe patients (n = 17)** | *p* |
| --- | --- | --- | --- |
| **Median ages (IQR)** | 45(37–55) | 56(44–71) | 0.02 |
| **Gender** |  |  |  |
| Female | 13(43%) | 7(41%) | >0.05 |
| Male | 17(57%) | 10(59%) | >0.05 |
| **BMI (IQR)** | 24.5 (23.8-24.9) | 24.6(23.7-25.1) | >0.05 |
| **Presenting symptoms** |  |  |  |
| Fever | 26(88%) | 14(82%) | >0.05 |
| Diarrhoea | 1(3%) | 0(0%) | >0.05 |
| Weak | 5(17%) | 7(41%) | >0.05 |
| Cough | 15(50%) | 13(76%) | >0.05 |
| Dyspnoea | 0(0%) | 8(47%) | >0.05 |
| **Chronic comorbidities** |  |  |  |
| None | 15(50%) | 7(41%) | >0.05 |
| Hypertension | 6(20%) | 6(35%) | >0.05 |
| Thalassemia | 1(3%) | 0(0%) | >0.05 |
| Subacute thyroiditis | 1(3%) | 0(0%) | >0.05 |
| Diabetes | 0(0%) | 0(0%) | >0.05 |
| asthma | 1(3%) | 1(6%) | >0.05 |
| Cerebral infarction | 0(0%) | 1(6%) | >0.05 |
| asthma | 0(0%) | 1(6%) | >0.05 |
| Hepatitis | 0(0%) | 1(6%) | >0.05 |

IQR: interquartile range.
