# Supplementary material for: Longitudinal Metabolomics Reveals Ornithine Cycle Dysregulation Correlates With Inflammation and Coagulation in COVID-19 Severe Patients
Source: Front Microbiol. 2021 Dec 3;12:723818. doi: 10.3389/fmicb.2021.723818 (PMC8678452; doi:10.3389/fmicb.2021.723818)
Supplement: Supplementary file 2 [file Table_2.docx]

Table S2 Basic information of 47 COVID-19 patients and 20 controls.

|  | **COVID-19 patients**  **(n=47)** | **Controls**  **(n = 20)** | ***p*** |
| --- | --- | --- | --- |
| **Median ages (IQR)** | 49(40-61) | 45(37–55) | >0.05 |
| **Gender** |  |  |  |
| Female | 20(43%) | 9(45%) | >0.05 |
| Male | 27(57%) | 11(55%) | >0.05 |
| **BMI** | 24.5 (23.8-25.0) | 24.4(23.7-24.8) | >0.05 |

IQR: interquartile range.
