# Supplementary material for: Longitudinal Metabolomics Reveals Ornithine Cycle Dysregulation Correlates With Inflammation and Coagulation in COVID-19 Severe Patients
Source: Front Microbiol. 2021 Dec 3;12:723818. doi: 10.3389/fmicb.2021.723818 (PMC8678452; doi:10.3389/fmicb.2021.723818)

Figure S1. The percentages of significantly regulated metabolites in different subclasses with the levels significantly different between COVID-19 patients and controls (FDR $p < 0.05$, VIP > 1.0). The classes of those metabolites are also shown in the right panel.

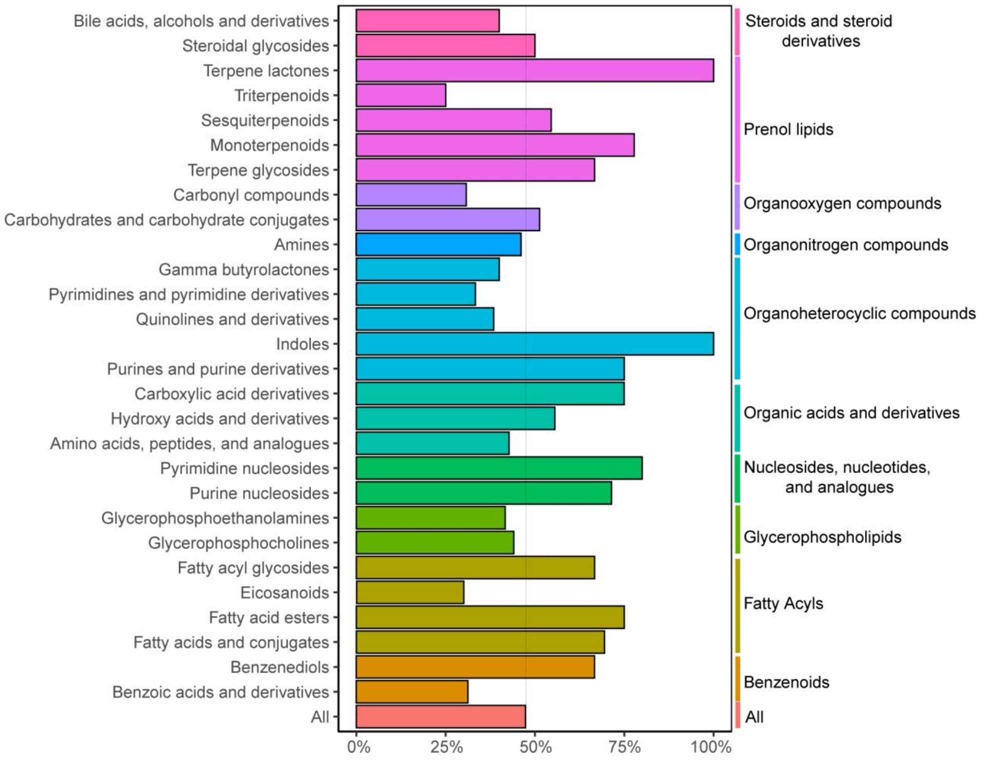

Supplement: Supplementary file 3 [file Image_1.pdf]
