# Supplementary material for: Longitudinal Metabolomics Reveals Ornithine Cycle Dysregulation Correlates With Inflammation and Coagulation in COVID-19 Severe Patients
Source: Front Microbiol. 2021 Dec 3;12:723818. doi: 10.3389/fmicb.2021.723818 (PMC8678452; doi:10.3389/fmicb.2021.723818)

Figure S2. The heat map showing the percentages of upregulated metabolites in the main subclasses from mild or severe patients at the four sampling times. The numbers of regulated metabolites in different subclasses are also marked in each square.

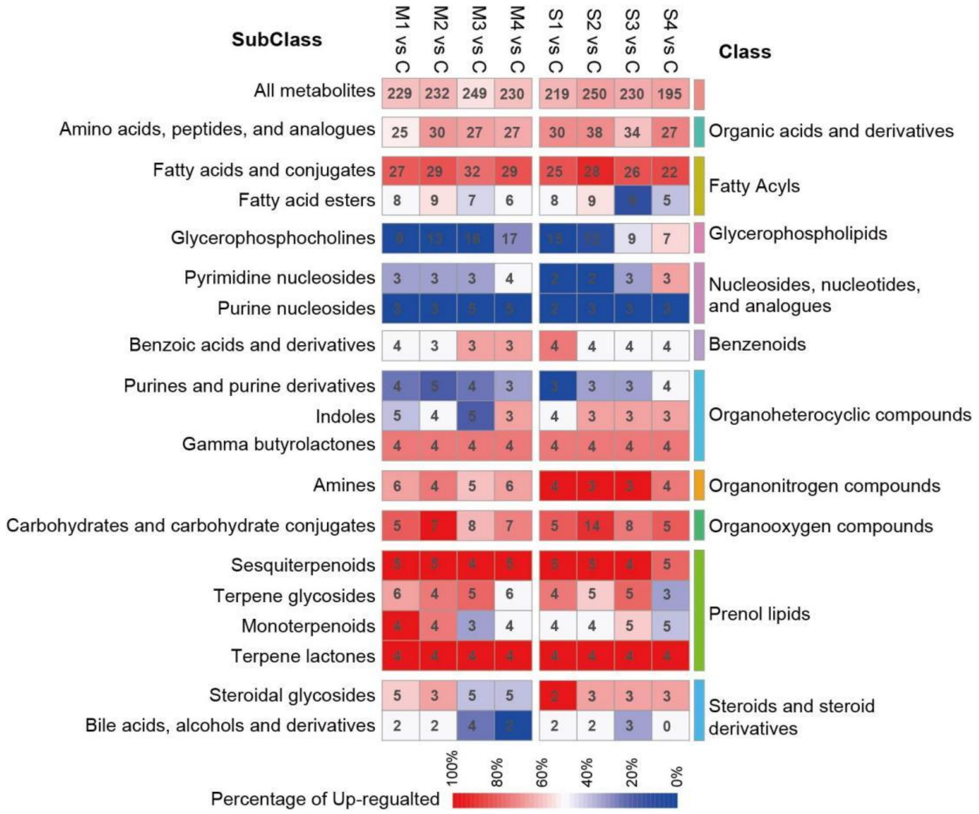

Supplement: Supplementary file 4 [file Image_2.pdf]
