# Supplementary material for: Longitudinal Metabolomics Reveals Ornithine Cycle Dysregulation Correlates With Inflammation and Coagulation in COVID-19 Severe Patients
Source: Front Microbiol. 2021 Dec 3;12:723818. doi: 10.3389/fmicb.2021.723818 (PMC8678452; doi:10.3389/fmicb.2021.723818)

Figure S3. The KEGG pathways with differential metabolites enrichment in mild or severe COVID-19 patients.

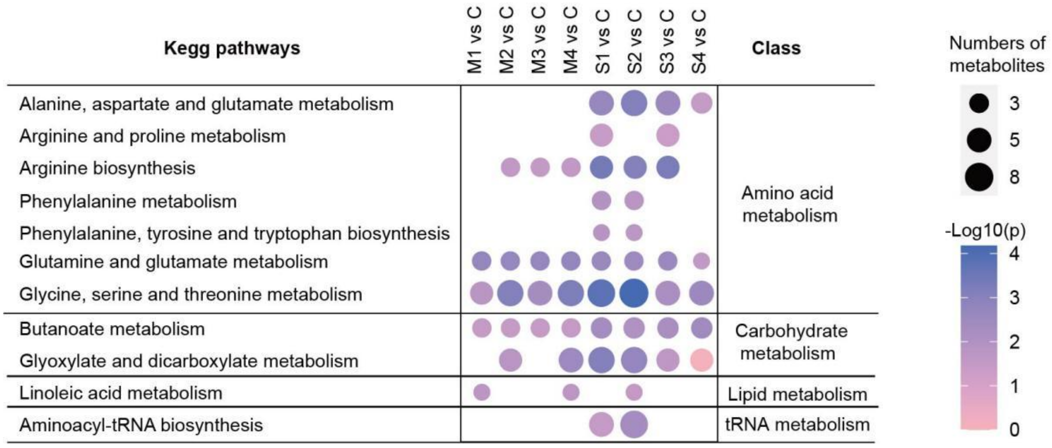

Supplement: Supplementary file 5 [file Image_3.pdf]
