# Supplementary material for: Longitudinal Metabolomics Reveals Ornithine Cycle Dysregulation Correlates With Inflammation and Coagulation in COVID-19 Severe Patients
Source: Front Microbiol. 2021 Dec 3;12:723818. doi: 10.3389/fmicb.2021.723818 (PMC8678452; doi:10.3389/fmicb.2021.723818)

Figure S5. The stage-specific metabolic signatures correlated with mild and severe patients

(A) The Venn diagram shows the numbers of significantly differential metabolites between mild and severe patients.

(B) The fold changes (FC) of five metabolites are significantly different at four stages between mild and severe patients. **, $p < 0.01$. *, $p < 0.05$.

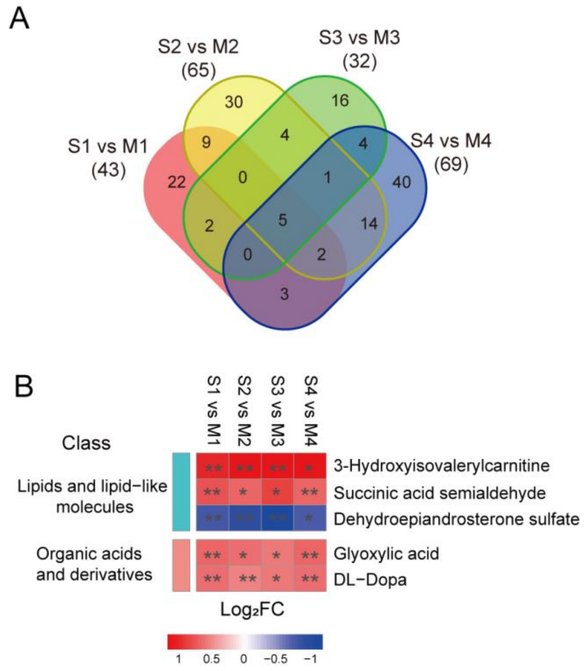

Supplement: Supplementary file 7 [file Image_5.pdf]
