# Supplementary material for: Longitudinal Metabolomics Reveals Ornithine Cycle Dysregulation Correlates With Inflammation and Coagulation in COVID-19 Severe Patients
Source: Front Microbiol. 2021 Dec 3;12:723818. doi: 10.3389/fmicb.2021.723818 (PMC8678452; doi:10.3389/fmicb.2021.723818)

Figure S6. (A) The levels of the cytokines from different sampling times (R1–3, 1–3 days before discharge) and associated *p* values for COVID-19 patients (mild, M; severe, S) compared with controls (C). (B, C) The levels of IL-6 or IL-7 and associated *p* values for severe patients compared with mild patients. (**, $p < 0.01$; *, $p < 0.05$; NS, not significant.)

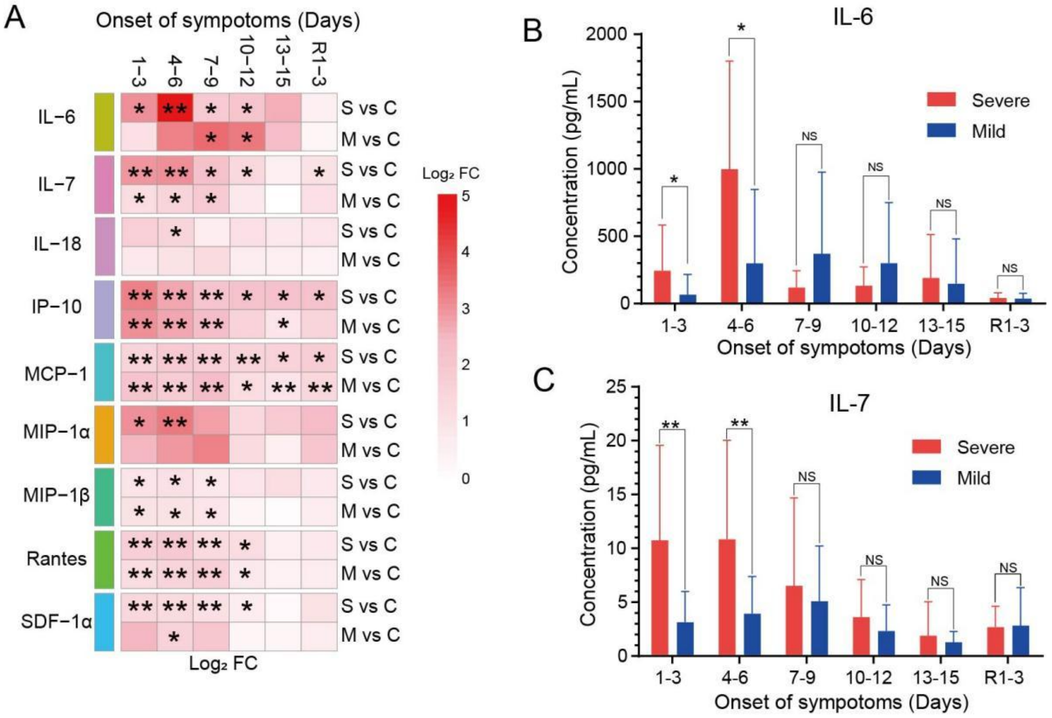

Supplement: Supplementary file 8 [file Image_6.pdf]
