# Supplementary material for: Longitudinal Metabolomics Reveals Ornithine Cycle Dysregulation Correlates With Inflammation and Coagulation in COVID-19 Severe Patients
Source: Front Microbiol. 2021 Dec 3;12:723818. doi: 10.3389/fmicb.2021.723818 (PMC8678452; doi:10.3389/fmicb.2021.723818)

Figure S7. The fold changes of biochemical parameters from different sampling times (R1–3, 1–3 days before discharge) and associated p values for severe patients compared with mild ones. **, $p < 0.01$; *, $p < 0.05$.

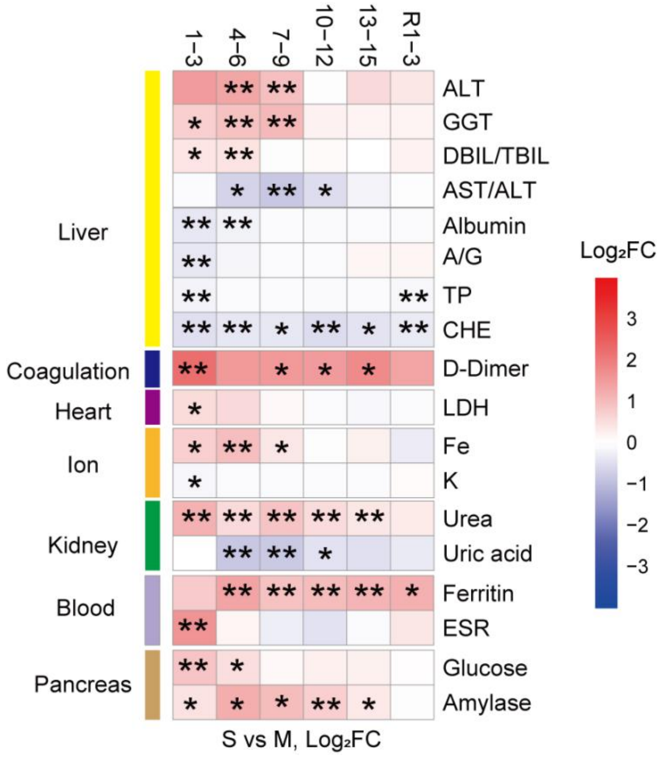

Supplement: Supplementary file 9 [file Image_7.pdf]
