# Supplementary material for: Longitudinal Metabolomics Reveals Ornithine Cycle Dysregulation Correlates With Inflammation and Coagulation in COVID-19 Severe Patients
Source: Front Microbiol. 2021 Dec 3;12:723818. doi: 10.3389/fmicb.2021.723818 (PMC8678452; doi:10.3389/fmicb.2021.723818)

Figure S8. The heat map of the correlation coefficients of different items in cluster 2 and representative metabolites in mild or severe samples.

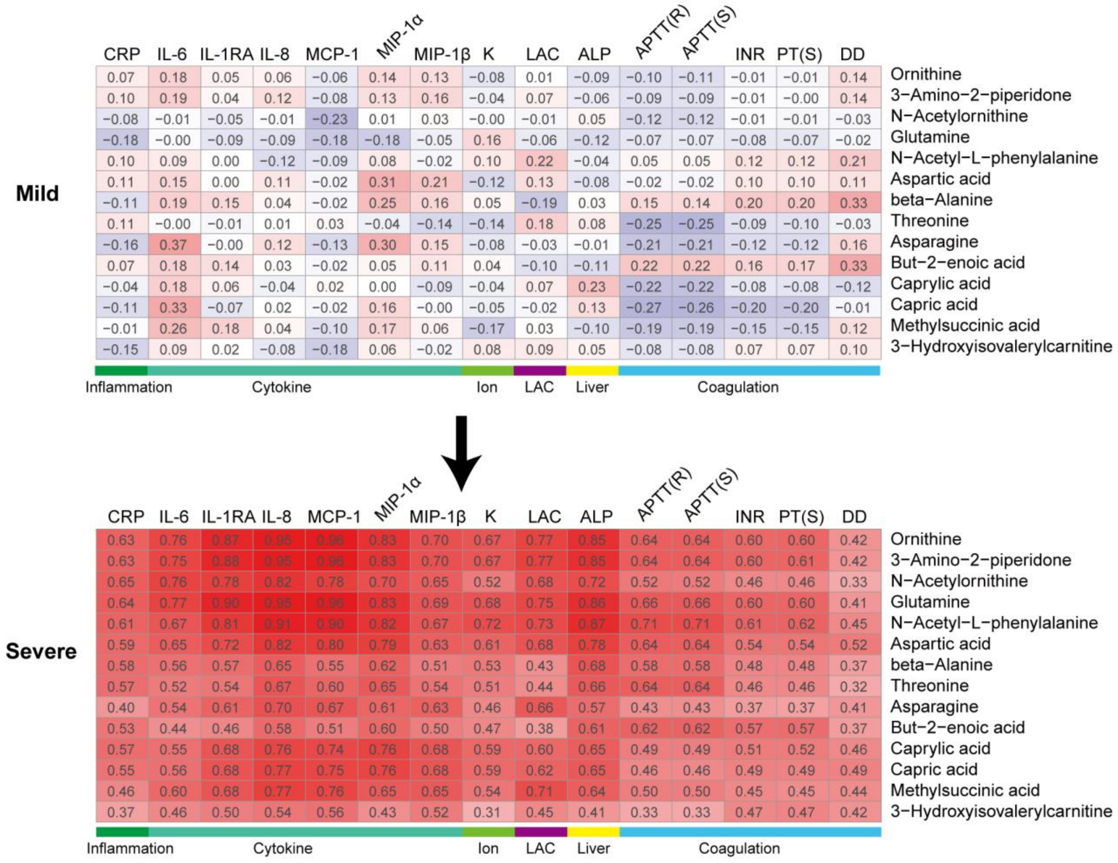

Supplement: Supplementary file 10 [file Image_8.pdf]
